# Spontaneous rupture of a splenic artery aneurysm causing acute abdomen in a 19-year-old male patient: a case report

**DOI:** 10.3389/fsurg.2023.1223271

**Published:** 2023-09-18

**Authors:** Yumna Njoum, Abdallah Deghles Barqawi, Mohammed Maree

**Affiliations:** ^1^ Faculty of Medicine, Al-Quds University, Jerusalem, Palestine; ^2^ Department of Surgery, Al-Makassed Hospital, Jerusalem, Palestine

**Keywords:** splenic artery, aneurysm, spontaneous rupture, hemoperitoneum, acute abdomen, splenectomy

## Abstract

**Introduction:**

A splenic artery aneurysm is considered an abnormal dilatation of the splenic artery layers greater than 1 cm in diameter. First described by Beaussier in 1770, it affects 1% of the population but carries a major risk for life-threatening complications of rupture in 3%–10% of cases regardless of its congenital or acquired etiology. The presentation is highly variable, from asymptomatic incidental discovery during routine imaging to aneurysmal rupture causing acute abdomen, massive gastrointestinal bleeding, and hemorrhagic shock.

**Case presentation:**

Herein, we present a 19-year-old male patient who presented with epigastric pain and abdominal rigidity associated with a moderate amount of free peritoneal fluid that was found to be a ruptured SAA after immediate laparoscopy, which was successfully managed with splenectomy.

**Conclusion:**

SAAs are a rare etiology of acute abdomen and hemorrhagic shock but have a very high risk of mortality even upon immediate intervention, requiring a very high level of vigilance and a low threshold for surgical intervention in unstable patients presenting with abdominal pain.

## Introduction

Although rare, with only 1% prevalence (1), a splenic artery aneurysm (SAA) is considered the third most common intra-abdominal aneurysm after the aortic and iliac artery aneurysms (2); however, due to the high risk of mortality when ruptured, it is of crucial importance to diagnose and manage it to prevent fatal outcomes, even though it occurs in only 10% of cases (1). Patients may be asymptomatic, but the rupture is catastrophic, with a mortality rate as high as 66% in aneurysms greater than 2 cm (3). SAAs can be congenital or acquired, and atherosclerosis, hypertension, trauma, and septic embolism play a role in their development. Pregnancy and multiparity are risk factors in the development of SAAs (4), and indeed, SAAs are frequent in female patients as the female-to-male ratio is 4:1 (3). Our 19-year-old male patient, who presented with sudden onset stabbing epigastric pain of 1-h duration, borderline blood pressure, and tachycardia associated with abdominal rigidity, was found to have free fluid on abdominal imaging. The patient was lucky and survived an acute abdomen and a hemorrhagic shock after nontraumatic spontaneous rupture of his previously undiagnosed SAA.

## Case presentation

A 19-year-old male patient with no significant medical or surgical history presented to the emergency department complaining of a 1-h history of sudden onset severe stabbing epigastric pain that then became generalized, radiated to both shoulders and back, associated with nausea but no vomiting, increased upon movement and while in the supine position but that was not related to food. The patient said they had no history of preceding trauma, vomiting episodes, hematemesis, melena, or changes in bowel habits. He also outlined that he had not had similar previous episodes, postprandial pain, alcohol consumption, and chronic analgesia ingestion. He worked as a construction worker and had no encounters with animals or sick contacts.

Upon examination, he was mildly hypotensive with a blood pressure of 106/72 and tachycardic. Capillary refilling time was 2 s; he was conscious and alert, lying in bed motionless in pain. He was pale and sweaty. Abdominal examination revealed voluntary guarding with generalized tenderness maximum on the epigastric area, questionable costovertebral angle tenderness, and hypoactive bowel sounds, and rectal examination revealed no blood or black tarry stools. Chest and heart examination revealed no abnormalities.

**Figure 1:**
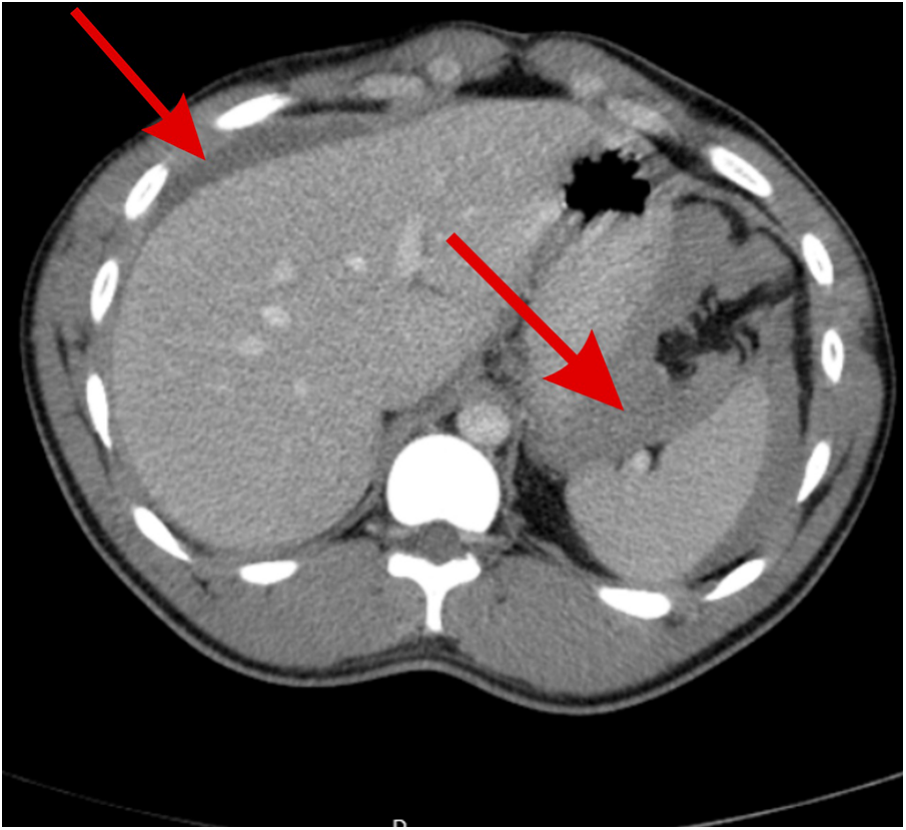
Abdominal CT scan showing free fluid of blood density around the spleen, between the spleen and stomach, and around the hepatic areas, indicating hemoperitoneum (red arrows).

**Figure 2:**
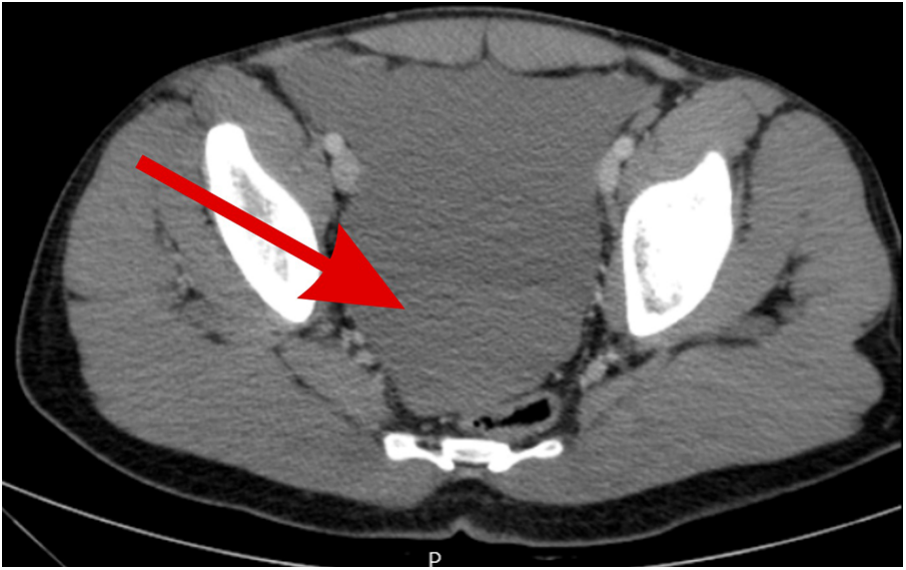
Pelvic CT scan showing free fluid in the pelvic area of fluid density indicating hemoperitoneum (red arrow).

Initial laboratory tests showed a hemoglobin level of 14 g/dL, a white blood cell count of 8 WBCs per microliter (8 × 10^9^/L), a platelet count of 254 platelets per microliter, normal liver enzymes, normal kidney and liver function tests, and normal lipase and amylase levels. Abdomen x-ray showed no bowel dilatation or air-fluid levels. Chest x-ray showed no subdiaphragmatic free air. Abdomen ultrasound showed normal internal organs of the abdomen except for moderate amounts of free fluid and no signs of cholecystitis, biliary dilatation, hydronephrosis, or splenic abnormalities. He underwent contrast-enhanced abdomen and pelvic computed tomography that showed moderate amounts of free fluid around the spleen, in the pelvis, between the stomach and spleen, and in Morison's pouch (Figures 1, 2); fluid density was >40 IU, suggestive of blood. No definite bleeding source was identified, so the patient was urgently transferred to the operating room for exploratory laparoscopy as a case of acute abdomen and hemoperitoneum; embolization was not attempted due to ongoing hemorrhage with peritoneal signs on examination and due to lack of definitive diagnosis. The exploration began laparoscopically, but the perisplenic, perihepatic, and pelvic areas were filled with bloodf and the source could not be identified, so it was converted to laparotomy through a midline incision where massive hemorrhage was encountered; 2 L of fresh blood was hardly suctioned, and multiple pads were required to control bleeding. The gastrocolic ligament was dissected widely, and a huge amount of coagulated blood was evacuated, allowing the identification of a bleeding arterial source that was clamped just behind the distal pancreas near the splenic hilum that was diagnosed to be an actively bleeding ruptured hilar splenic artery aneurysm measuring almost 2.5 cm. At the pancreatic upper border, the splenic artery was dissected and fixed with nonabsorbable sutures for the proximal control of the hemorrhage, effectively decreasing its volume. Hemorrhage was completely controlled via splenectomy *en bloc*. Running of the bowel and other internal organ examination was also done, and the result was normal with no other abnormalities. Hemostasis was secured, and the patient received two packed red blood cell units intra-operatively. Then, he was transferred to the intensive care unit for close monitoring with no immediate or early complications.

Postoperatively and after resuscitation, hemoglobin decreased to 11 g/dL, but white blood cell count, platelet count, lactic acid, and C-reactive protein were normal. The patient recovered well in 4 days with no events and was discharged home in excellent condition.

At 2-week follow-up after discharge, the patient reported no complaints, was vitally stable with normal physical examination, had a hemoglobin level of 12.7 g/dL, and had taken recommended postsplenectomy vaccinations including pneumococcal, meningococcal, and *Haemophilus influenzae* vaccines and annual vaccination as scheduled.

Pathologic examination of the resected specimen showed a 2.5-cm true splenic artery aneurysm with normal histology, excluding secondary pathological causes of the aneurysm.

## Discussion

A splenic artery aneurysm is an abnormal dilatation of the splenic artery layers greater than 1 cm in diameter (2). It was first described by Beaussier in 1770 but was never operated before 1920, 150 years since its discovery (1). In a large autopsy series that studied the incidence of SAA, it was found to range between only 0.01% and 0.2%, dramatically increasing to 10% in patients older than 60 years and in those with portal hypertension (5).

Symptomatic SAAs only occurred in 20% of cases, which presented with epigastric and left upper quadrant abdominal pain. Other nonspecific complaints included anorexia, nausea, or vomiting. The final diagnosis is nearly always obtained by abdominal imaging (6). Unfortunately, the catastrophic presentation is a spontaneous rupture of the SAA, which only occurs in 2%–10% of patients presenting complaints. The widespread availability of radiological services has reduced the rate of presenting with an acute aneurysmal rupture from 10% to approximately 3% of patients who present with sudden onset acute epigastric and right upper quadrant pain with radiation to shoulders associated with hemodynamic compromise (2). Our patient, who was unusually a male with no previous known risk factors, presented with a spontaneous nontriggered rupture of a 2-cm distal splenic artery aneurysm, fulfilling all the rare entities of this life-threatening event and surviving with no consequences.

Another rare presentation of SAA rupture is the double rupture phenomenon of the SAA that was described for the first time in 1930 also by Bockerman, in which aneurysms initially rupture into the lesser omental sac causing transient tamponade, during which patients are still stable, with mild or no symptoms. Six to ninety hours later, blood overflows into the peritoneum through the foramen of Winslow, leading to sudden hypovolemic shock and vascular collapse (7).

Another very uncommon presentation of SAA rupture causes secondary erosion of the SAA to an adjacent organ, leading to gastrointestinal hemorrhage (8).

Erosion of the aneurysm into the splenic vein also leads to arteriovenous fistula and portal hypertension and rarely results in mesenteric steal syndrome and small bowel ischemia (2).

Ultrasonography, pulsed Doppler, contrast-enhanced computed tomography scanning, magnetic resonance imaging, magnetic resonance angiography, and abdominal aortic arteriography are all radiographic modalities capable of diagnosing an asymptomatic aneurysm. Contrast-enhanced computed tomography scanning is the gold standard in detecting aneurysms and intra-abdominal bleeding. Until now, abdominal aortic angiography, however, is the most valuable modality of choice in localizing the bleeding source and assessing collateral vessels (9).

Regarding management, regardless of their dimensions, all symptomatic SAAs are believed to require treatment; patients with aneurysmal dimensions of 2 cm, pregnant or fertile patients, patients with portal hypertension, or candidates for liver transplantation patients also require management even if asymptomatic (10).

Excision, ligation, or revascularization are surgical alternatives, with or without splenectomy. Either an open or minimally invasive approach could be used. Those who are not candidates for surgery and those in elective settings are typically given the endovascular option, which includes various techniques such as coil embolization, covered stents placement, gluing, plug deployment, and endoluminal thrombin, polyvinyl alcohol, particles, or gel foam injection. However, surgical intervention remains the gold standard treatment in most centers, especially in cases of rupture (1, 10).

## Conclusion

This study has described a case of a male patient who presented with acute abdomen, signs of shock, and evident free fluid on imaging that was later found to have a ruptured SAA. The rare etiology and rarer presentation in an unusual gender and age group, highlight the importance of having a very high level of awareness about SAAs as a potential cause of bleeding and a low threshold for more advanced imaging modalities for diagnosing conditions with very high mortality and an even lower threshold for intervention in which mortality rates dramatically increase with every minute of delay.

## Patient's perspective

I am grateful for the opportunity to share my medical journey through this case report. It is my hope that by sharing my experience, others may gain valuable insights and knowledge that can contribute to the understanding and treatment of similar conditions. I want to express my sincere appreciation to the healthcare professionals involved in my care, whose expertise and compassion made a significant impact on my well-being. Their dedication and commitment to providing quality healthcare have been truly remarkable. Lastly, I extend my gratitude to the authors and researchers involved in the preparation of this case report for their dedication to advancing medical knowledge. Together, we can contribute to improving patient care and outcomes for future individuals facing similar medical challenges.

## Data Availability

The original contributions presented in the study are included in the article/Supplementary Material, further inquiries can be directed to the corresponding author.
